# Clinical, Radiological, and Outcome Characteristics of Acute Pulmonary Embolism: A 5-year Experience from an Academic Tertiary Center

**DOI:** 10.5339/qmj.2022.1

**Published:** 2022-05-09

**Authors:** Wanis H Ibrahim, Shaikha D Al-Shokri, Musa S Hussein, Lana M Abu Afifeh, Gowri Karuppasamy, Jessiya V Parambil, Farras M Elasad, Mohammed E Faris, Mohamed S Abdelghani, Ahmed Abdellah, Antoun Kamel, Hafedh Ghazouani, Mushtaq Ahmad, Aisha Aladab, Mohammed I Danjuma, Tasleem Raza

**Affiliations:** ^1^Hamad General Hospital, Doha, Qatar E-mail: wibrahim@hamad.qa; ^2^Metro Health Medical Center, Cleveland, Ohio, United States; ^3^College of Medicie, Qatar University, Doha, Qatar; ^4^Milton Keynes University Hospital NHS, United Kingdom

**Keywords:** Venous thromboembolism, pulmonary embolism, clinical features, mortality

## Abstract

Background: Acute pulmonary embolism (PE) is a common and potentially life-threatening condition. This comprehensive study from a Gulf Cooperation Council (GCC) country aimed to evaluate the clinical, radiological, and outcome characteristics associated with acute PE.

Methods: This retrospective observational study analyzed data of patients with confirmed acute PE who were admitted to the largest academic tertiary center in the State of Qatar from January 1, 2014, to December 31, 2018. Data on the clinical presentation, radiologic, and echocardiographic findings, as well as outcomes were collected.

Results: A total of 436 patients were diagnosed with acute PE during the study period (male, 53%). Approximately 56% of the patients were < 50 years old at presentation, with a median age of 47 years. In approximately 69% of cases, the PE occurred outside the hospital. The main associated comorbidities were obesity (34.6%), hypertension (29.4%), and diabetes (25%). Immobilization (25.9%) and recent surgery (20.6%) were the most common risk factors. The most frequent presenting symptom was dyspnea (39.5%), and the most frequent signs were tachycardia (49.8%) and tachypnea (45%). Cardiac arrest was the initial presentation in 2.2% of cases. Chest X-ray findings were normal in 41%. On computed tomography pulmonary angiography (CTPA), 41.3% of the patients had segmental PE, 37.1% had central PE, and 64.1% had bilateral PE. The main electrocardiographic (ECG) abnormality was sinus tachycardia (98%). In patients who underwent echocardiography, right ventricular (RV) enlargement was the main echocardiographic finding (36.4%). Low-, intermediate-, and high-risk PE constituted 49.8%, 31.4%, and 18.8% of the cases, respectively. Thrombolysis was prescribed in 8.3% of the total and 24.4% of the high-risk PE cases. Complications of PE and its treatment (from admission up to 6 months post-discharge) included minor bleeding (14%), major bleeding (5%), PE recurrence (4.8%), and chronic thromboembolic pulmonary hypertension (CTEPH) (5%). A total of 15 (3.4%) patients died from PE.

Conclusions: Acute PE can manifest with complex and variable clinical and radiological syndromes. Striking findings in this study are the younger age of acute PE occurrence and the low PE-related mortality rate.

## Introduction

Venous thromboembolism (VTE) is a common disease that has serious consequences including reduced survival, substantial healthcare costs, and high recurrence rate. Currently, VTE encompasses deep venous thrombosis (DVT) of the legs or pelvis and its more serious sequelae, PE ^1^. The pathophysiology of PE is complex and involves interactions between acquired or inherited predispositions to thrombosis and various risk factors. The ultimate effects of impaired hemodynamics, gas exchange, and lung mechanical capacity play a pivotal role in the high-risk PE-related death.^2^ The obstruction of the pulmonary bed due to PE can result in acute RV failure, malignant arrhythmias, shock, or even sudden cardiac death. In the long run, PE can result in CTEPH-, which is defined as mean pulmonary artery pressure ≥ 25 mmHg with normal capillary wedge pressure persisting 6 months after acute PE, and PE recurrence.^3,4^ The incidence rates of PE (with or without DVT) and DVT alone (without PE) range from 29 to 78, and 45 to 117, per 100,000 person-years, respectively. The incidence rates increase markedly with age for men and women and vary with the geographical distribution.^1,5–9^ Despite the identification of the risk factors and predictors of VTE recurrence and the availability of effective primary and secondary prophylaxes, the incidence of VTE is generally constant or even increasing.^1^ Regardless of the recent decline, high PE mortality rates are still observed even in developed countries. Between 2013 and 2015, PE accounted for 8–13 and 2–7 per 1000 deaths in women and men aged 15–55 years, respectively, in the European region.^10^ PE can present with various clinical syndromes, ranging from being asymptomatic to shock or sudden death. Previous studies found that antemortem PE diagnosis was rarely suspected among patients who died of PE.^11,12^ A high index of suspicion based on various clinical and laboratory characteristics should be maintained to diagnose PE and reduce the likelihood of inadvertently discarding the diagnosis.^12^ In this comprehensive study, we report the clinical, radiological, and outcome characteristics of 436 patients with confirmed acute PE.

## Methods

### Study design, setting, and participants

This retrospective descriptive study included adult patients (aged >14 years) who were admitted to Hamad General Hospital (the largest tertiary hospital in the State of Qatar) for acute PE from January 1, 2014, to December 31, 2018. In the Qatari health system, patients aged ≤ 14 years are classified as “pediatric” and normally admitted to pediatric hospitals and therefore excluded from this study.

### Data collection and study definitions

Patients were identified using the registers of the services. Patients’ electronic medical records were extensively searched to confirm the consistency and completeness of the information. Data related to sociodemographic factors, clinical presentation, comorbidities, and risk factors of acute PE, as well as the results of chest X-ray, ECG, echocardiography, and CTPA, were extracted from the electronic records and recorded in a structured data collection sheet. PE was diagnosed based on the presence of a thrombus in the pulmonary artery or any of its branches using CTPA, direct visualization of the emboli in the right heart chambers or central pulmonary arteries via echocardiography, or high-probability V/Q (lung scintigraphy) scan along with high clinical probability for PE. The following risk factors were investigated: obesity (body mass index [BMI] of 30 kg/m^2^), cancer, previous VTE, recent long trip, pregnancy/use of oral contraceptive pills, recent surgery, and immobilization. Medications used for the initial and maintenance treatment of PE and PE outcomes from the time of diagnosis up to 6 months post-discharge were also reported. We used the 2019 European Society of Cardiology (ESC) definitions to classify PE severity and risk of early death.^13^ High-risk PE was defined by the presence of one of the following clinical presentations: cardiac arrest, obstructive shock (systolic blood pressure [BP] < 90 mmHg or vasopressors required to achieve a BP ≥ 90 mmHg despite an adequate filling status, in combination with end-organ hypoperfusion), or persistent hypotension (systolic BP < 90 mmHg or a systolic BP drop ≥ 40 mmHg for >15 min, not caused by new-onset arrhythmia, hypovolemia, or sepsis). Intermediate-risk PE was defined by the presence of one of the following: signs of RV dysfunction on transthoracic echocardiography (or CTPA) or elevated cardiac biomarker levels in the absence of hemodynamic instability. Low-risk PE was defined by the absence of any of the features in the high- or intermediate-risk category.^13^ Major bleeding was defined as the occurrence of any of the following: (1) fatal bleeding; (2) symptomatic bleeding in a critical area or organ, such as intracranial, intraspinal, intraocular, retroperitoneal, intra-articular or pericardial, or intramuscular with compartment syndrome; and/or (3) bleeding causing a fall in hemoglobin level of ≥ 2 g/dL or leading to transfusion of ≥ 2 units of whole blood or red cells.^14^

### Statistical analysis

Qualitative and quantitative data were expressed as the frequency with percentage and mean ±  standard deviation with median and range. Descriptive statistics were used to summarize the demographic and all other clinical characteristics of the participants. The crude annual incidence of PE was calculated based on the adult population (aged ≥ 18 years). A two-sided *p* < 0.05 was considered significant. We used STATA version 12.0 (StataCorp, College Station, TX, USA) for exploratory data analysis and descriptive statistics.

### Ethical approval

The study was approved by the Medical Research Center at Hamad Medical Corporation (Approval no: MRC-01-19-059).

## Results

During the study period, 436 patients were diagnosed with acute PE and met the inclusion criteria (432 patients diagnosed by CTPA, three by V/Q scan criteria, and one by visualization of clots on echocardiography). The crude incidence rate was 88 cases per 100 000 adult populations annually. Male patients constituted 53% of the total patients. The majority of the patients were non-Qatari nationals. The median age at diagnosis was 47 (interquartile range [IQR] 35–61) years, and participants aged 30–49 years were the most represented (43.3%). The majority (68.6%) of PE events occurred outside the hospital (Table 1). Obesity (34.6%), hypertension (29.4%), and diabetes (25%) were the most common associated comorbidities (Table 2). The risk factors of VTE found in this study are presented in Table 3. Immobilization (25.9%), recent surgery (20.6%), active cancer (12.4%), and recent long trip (11.9%) were the most common risk factors. Table 4 summarizes the symptoms and signs of PE at presentation. Dyspnea (39.5%) was the most common presenting symptom. Nine (2.2%) patients presented with cardiac arrest, whereas syncope was seen in 19 (4.7%) patients. The most common signs were tachycardia (49.8%) and tachypnea (45%). Clinical signs of DVT were observed in 69 (15.8%) patients, and the mean oxygen (O_2_) saturation at presentation was 95% (min–max, 60%–100%). Based on the 2019 ESC classification of PE severity,^13^ 49.8%, 31.4%, and 18.8% of the patients had low-, intermediate-, and had high-risk PE, respectively. A normal X-ray finding was the most common finding on chest radiography (41%), whereas the most common abnormalities were pleural effusion (25.4%) and atelectasis (21.6%). On CTPA, 41.3% had segmental PE, 37.1% had central PE, 64.1% had bilateral PE, and 23.8% had unilateral right-sided PE (Table 5). On ECG, 98% of the patients had sinus tachycardia, 42.2% had ST-T wave changes, and 30.2% had S1-Q3-T3 Pattern. RV enlargement was the most common finding in patients who underwent echocardiography (36.4%) (Table 6). Low molecular weight heparin (LMW heparin) was the most commonly prescribed initial therapy (66.5%). Thrombolytic therapy was administered in 36 (8.3%) of the total and 24.4% (20/82) of the patients with high-risk PE. Direct oral anticoagulants (DOAC) and warfarin were the most commonly prescribed maintenance treatment (Table 7). Minor bleeding from medications was the most frequent complication (14%). CTEPH and PE recurrence were observed in 5.0% and 4.8%, respectively. A total of 15 (3.4%) patients died from PE during the study period (male = 9, female = 6, Qatari nationals = 3, non-Qatari = 12, mean age = 53 ± 19 years, and median age [IQR] = 54 [36–70] years (Table 8).

## Discussion

This study represents the largest cohort of patients with a confirmed diagnosis of PE in the State of Qatar and shows the key findings concerning the clinical characteristics and outcomes of patients with PE. One of the most striking findings is the younger age of acute PE occurrence. While clinical data from Western countries indicate that most PE cases occur at age 60–70 years, autopsy data revealed the highest incidence among individuals aged 70–80 year.^3^ Younger age at PE occurrence has also been reported in other GCCcountries. Algahtani et al. reported a mean age of 45.1 years.^15^ Similar young age was also reported from a GCC country concerning DVT occurrence.^16^ Nevertheless, such young age of PE occurrence in the State of Qatar and other GCC countries should be interpreted with caution as it probably reflects the population structure in these countries. Reliance on the young labor force from Asia and other parts of the world for economic and industrial developments has resulted in changes in the demographic composition of these countries. For example, approximately 98% of the population in the State of Qatar is below the age 60 years, with the vast majority being expatriates and a male-to-female ratio of 3:1.^17^ About 31% of PE incidents in the present study occurred in hospitals, and the majority of these occurred in surgical wards. Furthermore, we found recent hospitalization for surgical reasons as an important risk factor for PE. VTE remains the most common preventable cause of death in hospitalized patients. Pharmacologic prophylaxis has been proven to reduce the risk of PE by 75% and 57% in general surgical and medical patients, respectively.^18–20^ Although any surgery carries a risk for VTE development, surgical procedures that carry the highest risk of postoperative VTE development include hip and knee arthroplasty, invasive neurosurgical procedures, and major vascular procedures.^21^ Despite the growing volume of evidence supporting the use of thromboprophylaxis, several studies have reported that nearly half of the patients undergoing major surgery or hospitalized for medical illnesses do not receive appropriate antithrombotic prophylaxis.^18,19,22^ In agreement with other studies, the present study presented that hypertension and obesity as the main associated comorbidities with immobilization and recent surgery are common risk factors for PE.^23^ In the present study, the most frequent presenting symptom was dyspnea, whereas the most frequent signs were tachycardia and tachypnea. Syncope and cardiac arrest were the initial presentations in 4.7% and 2.2% of cases. Clinical signs of lower-limb DVT were observed in approximately 16% of cases. The EMPEROR study (a national, prospective, multicenter, observational registry from the USA) reported that the most common presenting signs and symptoms were dyspnea at rest (50%), pleuritic chest pain (39%), dyspnea with exertion (27%), and extremity swelling suggestive of DVT (24%).^23^ Atypical presentation such as syncope is recently recognized as an important manifestation of acute PE. Nevertheless, the current international guidelines barely focused on establishing a diagnostic workup for PE in patients with syncope.^24^ The prevalence of syncope among patients with acute PE ranges from 1% to 20%.^25,26^ Recent studies have also linked syncope with high short-term mortality in patients with PE.^25^ The three major pathophysiological mechanisms proposed for the development of syncope in acute PE are acute right heart failure, hemodynamically unstable dysrhythmia (such as bradycardia or tachycardia), and vasovagal reflex. Together, these data suggest that PE should be a diagnostic consideration in patients presenting with syncope. The occlusion of >50% of the pulmonary vascular bed can cause acute RV failure, impaired left ventricular filling, tachycardia, hypotension, and reduced cerebral perfusion.^26^ In the present study, chest radiograph findings were normal in 41% of patients with acute PE. Chest radiography is not useful in the diagnosis of PE, per se, but it is useful in excluding other causes of acute chest pain such as pneumonia, pulmonary edema, or pneumothorax.^27^ Some radiographic abnormalities may have relatively high specificity for PE. These include the Fleischner sign (an enlarged pulmonary artery secondary to pulmonary hypertension or distension of the vessel by a pulmonary embolus), Westermark sign (regional oligemia from PE), and Hampton hump sign (a peripherally located wedge-shaped opacity in patients with pulmonary infarction).^28^ In the present study, the Fleischner sign, Westermark sign, and Hampton hump sign occurred in 1.6%, 16.4%, and 12.6% of the patients, respectively. Low-, intermediate-, and high-risk PE constituted 49.8%, 31.4%, and 18.8% of the cases, respectively, in the present study. In a study from India, massive PE was diagnosed in 28.5% of the patients, submassive in 60%, and minor PE in 11.5%.^29^ Some studies from Western countries have reported a low massive PE rate of 3%. However, such studies were criticized for utilizing only the presence of hypotension as a defining point of massive PE and recruiting patients with PE from emergency department registries.^23^ Nevertheless, the high prevalence of low-risk PE in the present study should be interpreted with caution, as not all patients underwent echocardiography. In a recent systematic review and meta-analysis of 21 cohort studies with a total of 3295 patients with “low-risk” PE based on clinical criteria, 34% of these patients were reported to have signs of RV dysfunction on echocardiography or CTPA which was associated with early mortality.^13,30^ Therefore, several patients who were diagnosed with low-risk PE based on clinical criteria may have intermediate-risk PE if echocardiography was performed. One of the very striking findings in the present study is the low mortality rate compared with the figures from many developed countries. Reports from Germany, Japan, Italy, and certain parts of the USA reported mortality rates of 17%, 14%, 10%, and 12%, respectively.^31–34^ Nevertheless, a few studies that have included only outpatients with PE also reported low mortality rates.^23^ The low mortality rate in the present study probably reflects several factors: (1) younger age of the study population, (2) availability of rapid CTPA scanning and wide applicability of suspected PE protocol in this academic tertiary center, and (3) increased use of LMW heparin, which may have affected mortality.^23^

To the best of our knowledge, the present study is the largest and most comprehensive from a GCC country to document the clinical, radiological, and outcome profiles associated with acute PE.^15,35,36^ Furthermore, the study was conducted in a setting where all noninvasive imaging tests are available and easily accessed, which led to the confirmation of PE diagnosis in all studied cases and was reflected by the low mortality rate.

Nevertheless, the present study has important limitations. First, besides the inherent limitations of retrospective studies, this study was conducted in a single center. Second, echocardiographic evaluation and some important laboratory tests were not performed in all patients with PE. This could have resulted in an underestimation of the true prevalence of intermediate-risk PE. Third, because of the lack of clear documentation, we could not ascertain the reasons for the low rate of thrombolysis administration (8.3% of total and 24.4% of the high-risk PE cases) and the reasons for the underutilization of thromboprophylaxis in in-hospital PE.

## Conclusions

Acute PE can manifest with complex and variable clinical and radiological syndromes. Striking findings in this study are the younger age of acute PE occurrence and the low mortality from PE compared with reports from many Western countries. Nevertheless, the high percentage of the young population in the country is a plausible contributory factor to such findings. Despite the availability of effective thromboprophylaxis and clear prophylaxis guidelines, PE still occurs in the hospital settings, particularly in the surgical wards. Future prospective studies should focus on the echocardiographic risk stratification of PE and timely administration of thrombolysis in high-risk cases.

## Data Availability Statement

The data that support the findings of this study are available from the corresponding author upon reasonable request.

## Funding

The publication of this article was funded by the Medical Research Center at Hamad Medical Corporation.

## Figures and Tables

**Table 1:** General characteristics of the study population

Variables	Frequency (N)	(%)

Gender (n = 436)		

Male	231	53.0%

Female	205	47.0%

Nationality (n = 436)		

Qatari	117	26.8%

Non-Qatari	319	73.2%

Age (years) (n = 436)		

Median age (years) (IQR*)	47(35–61)	

< 30	55	12.6%

30–39	96	22.0%

40–49	93	21.3%

50–59	74	17.0%

60–69	52	11.9%

≥ 70	66	15.1%

Where did PE happen? (n = 436)		

Outside hospital	299	68.6%

In-hospital	137	31.4%

In-hospital PE (place) (n = 137)		

Surgical ward	89	65.0%

Medical ward	48	35.0%

VTE* prophylaxis used before development of in-hospital PE (n = 79)		

Unfractionated heparin	14	17.7%

LMW Heparin**	23	29.1%

Pneumatic compressions	7	8.9%

No VTE prophyalxis	32	40.5%

Others (warfarin)	3	3.8%


*VTE, venous thromboembolism

**LMW, low molecular weight

**Table 2:** Baseline comorbidities of the study population (n = 436)

Variables	N	%

Obesity (BMI>30*)	151	34.6%

Hypertension	128	29.4%

Diabetes	109	25.0%

Smoking	55	12.6%

Ischemic heart disease	31	7.1%

Stroke	24	5.5%

Alcohol intake	18	4.1%

Congestive heart failure	17	3.9%

Asthma	17	3.9%

OSA**	12	2.8%

COPD***	11	2.5%

Interstitial lung disease	5	1.1%


*BMI, body mass index

**OSA, obstructive sleep apnea

***COPD, chronic obstructive pulmonary disease

**Table 3:** Risk factors for PE (n = 436)

Variables	N	%

Family history of thrombosis Recent long trip Immobilization Recent hospitalization for surgery Recent hospitalization for a medical reason Thrombophilia Recent fracture Active cancer Pregnancy Use of OCP*	16 52 113 90 48 33 55 54 14 23	3.7% 11.9% 25.9% 20.6% 11.0% 7.6% 12.6% 12.4% 3.2% 5.3%


*OCP, oral contraceptive pills

**Table 4:** Clinical presentation and severity of PE

Chief presenting symptom (n = 405)		

Dyspnea	160	39.5%

Chest pain	123	30.4%

Syncope	19	4.7%

No symptoms	18	4.4%

Palpitations	11	2.7%

Dizziness	10	2.5%

Hemoptysis	10	2.5%

Cough	9	2.2%

Cardiac arrest	9	2.2%

Arrhythmia	1	0.2%

Others (abdominal pain/reduced oral intake/fever)	35	8.6%

Duration of symptoms (n = 369)		

< 1 h	38	10.3%

1–2 h	14	3.8%

2–12 h	3	0.8%

12–24 h	2	0.5%

1–7 days	270	73.2%

>7 days	42	11.4%

Signs of PE (n = 436)		

Tachypnea (PR>20*)	196	45.0%

Tachycardia (RR>100**)	217	49.8%

JVP*** distension	5	1.1%

Pitting leg edema	35	8.0%

Parasternal heave	4	0.9%

S3 gallop	2	0.5%

Clinical signs of DVT $	69	15.8%

Oxygen saturation%: Mean (min–max)	95 (60–100)	

PE severity classification (n = 436)		

Low-risk PE	217	49.8%

Intermediate-risk PE	137	31.4%

High-risk PE	82	18.8%


*PR, pulse rate

**RR, respiratory rate

***JVP, jugular venous pressure

$ DVT, deep vein thrombosis

**Table 5:** Radiologic and laboratory findings

Variables	N	%

Findings on chest radiograph (n = 366)		

Normal chest X-Ray	150	41.0%

Pleural effusion	93	25.4%

Atelectasis	79	21.6%

Oligemia (Westermark sign)	60	16.4%

Pleural-based opacity (Hampton hump)	46	12.6%

Prominent central pulmonary artery (Fleischner sign)	6	1.6%

Location of the PE on CTPA* (n = 426)		

Central	158	37.1%

Lobar	60	14.1%

Segmental	176	41.3%

Subsegmental	32	7.5%

Lung involved on CTPA (n = 432)		

Bilateral PE	277	64.1%

Left-sided PE	52	12.0%

Right-sided PE	103	23.8%

Laboratory findings Median (Min–Max)		

Troponin T (N: 3–15 ng/L) (n = 213)	24 (10–103.5)	

NT-Pro-BNP** (N: 0–376 pg/mL) (n = 224)	2063 (1200–3575)	

D-dimer (N: 0–0.49 mg/L) (n = 279)	5.15 (2.7–10.64)	


*CTPA, computed tomography pulmonary angiography

**NT-Pro-BNP, NT-proB-type natriuretic peptide

**Table 6:** Echocardiographic and electrocardiographic findings

Echocardiographic findings (n=327)		

Right ventricular dysfunction	85	26.0%

Right ventricular enlargement	119	36.4%

Right ventricular hypokinesis	67	20.5%

Tricuspid regurgitation	112	34.2%

RVSP (mmHg) (n = 279)		

Mean	42 ± 17	

RVSP ≥ 40 *	158 56.6%	

RVSP>40	121 43.4%	

ECG abnormalities (n = 192)		

Tachycardia	188	97.9%

Right Axis deviation	23	12.0%

Positive R in V1	22	11.5%

T inversion in V1–V4	57	29.7%

ST segment depression in V1–V4	24	12.5%

S1-Q3-T3 Pattern	58	30.2%


*RVSP, right ventricular systolic pressure

**Table 7:** Treatment received (initial and maintenance)

Variables	N	%

Initial treatment (n = 436)		

Thrombolysis	36	8.3%

Thrombectomy	2	0.5

Unfractionated heparin	66	15.0%

LMW* heparin	290	66.5%

DOAC**	20	4.6%

Fondaparinux	1	0.2%

IVC filter ***	21	4.8%

Maintenance treatment (n = 380)		

Warfarin	166	43.7%

DOAC	167	43.9%

LMW heparin	47	12.4%


*LMW, low molecular weight

**DOAC, directly acting oral anticoagulan

***IVC, inferior vena cava

**Table 8:** Complications at follow-up (0–6 months) (n = 436)

Variables	N	%

Mortality	15	3.4%

PE recurrence	21	4.8%

CTEPH*	22	5.0%

Minor bleeding	61	14.0%

Major bleeding	22	5.0%


*CTEPH: chronic thromboembolic pulmonary hypertension
